# Correction: Shin et al. Optimized 3D Bioprinting Technology Based on Machine Learning: A Review of Recent Trends and Advances. *Micromachines* 2022, *13*, 363

**DOI:** 10.3390/mi16040415

**Published:** 2025-03-31

**Authors:** Jaemyung Shin, Yoonjung Lee, Zhangkang Li, Jinguang Hu, Simon S. Park, Keekyoung Kim

**Affiliations:** 1 Biomedical Engineering Graduate Program, Schulich School of Engineering, University of Calgary, Calgary, AB T2N 1N4, Canada; jaemyung.shin@ucalgary.ca (J.S.); zhangkang.li@ucalgary.ca (Z.L.); jinguang.hu@ucalgary.ca (J.H.); 2 Department of Mechanical and Manufacturing Engineering, Schulich School of Engineering, University of Calgary, Calgary, AB T2N 1N4, Canada; yoonjung.lee@ucalgary.ca (Y.L.); simon.park@ucalgary.ca (S.S.P.); 3 Department of Chemical and Petroleum Engineering, Schulich School of Engineering, University of Calgary, Calgary, AB T2N 1N4, Canada

## 1. Error in Figure 3

In the original publication [1], the authors did not receive copyright permission for subfigure B of Figure 3. Figure 3 has been corrected, and the subfigure in question has been replaced with the new one created by the authors.

**Figure 3:**
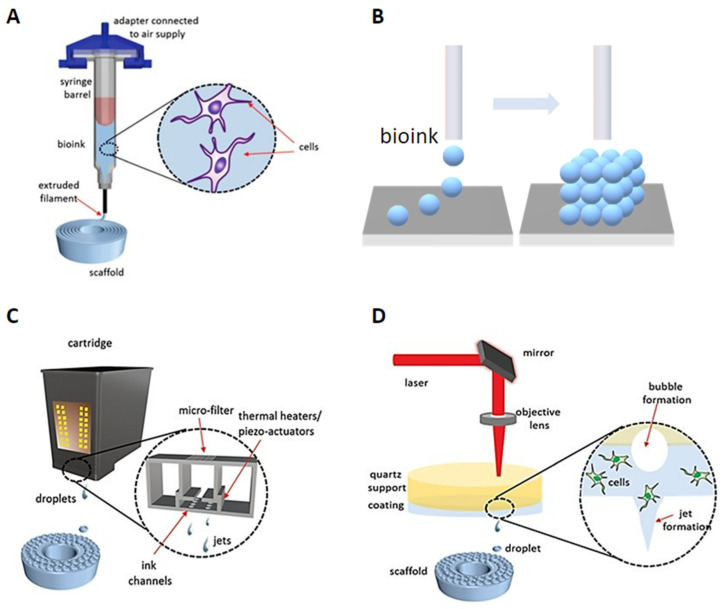
Modalities for bioprinting technologies. (**A**) Extrusion-based bioprinting. (**B**) Scaffold-free bioprinting. (**C**) Inkjet bioprinting. (**D**) Laser-induced forward transfer bioprinting (adapted from [32] with permission).

In the original paper [1], Reference 67 was incorrectly cited in the caption of Figure 4. The correct citation is Reference 66. The correct reference that should be cited in Figure 4’s caption appears below:

66. Lee, J.; Oh, S.J.; An, S.H.; Kim, W.-D.; Kim, S.-H. Machine learning-based design strategy for 3D printable bioink: Elastic modulus and yield stress determine printability. *Biofabrication* **2020**, *12*, 035018.

With this correction, the order of references does not need to be adjusted. 

The authors state that the scientific conclusions are unaffected. The correction was approved by the Academic Editor. The original publication has also been updated.

## References

[1] Shin J., Lee Y., Li Z., Hu J., Park S.S., Kim K. (2022). Optimized 3D Bioprinting Technology Based on Machine Learning: A Review of Recent Trends and Advances. Micromachines.

